# Minimum Hepatic Encephalopathy

**DOI:** 10.4103/1319-3767.70628

**Published:** 2010-10

**Authors:** Sourabh Aggarwal

**Affiliations:** University College of Medical Sciences, New Delhi, India. E-mail: drsourabh79@gmail.com

Sir,

I read the article ‘Predictors of minimal hepatic encephalopathy in patients with cirrhosis’ by Sharma *et al*. with interest.[1] The authors have conducted an interesting study, but a few points need to be highlighted. Studies done in the past have also shown the correlation between EEG (electroencephalography) and psychometric hepatic encephalopathy score in up to 73% of the cases and can be used as a non-invasive modality to diagnose cases of minimum hepatic encephalopathy in settings where it is possible.[2]
